# The Integral Role of RNA in Stress Granule Formation and Function

**DOI:** 10.3389/fcell.2021.621779

**Published:** 2021-05-20

**Authors:** Danae Campos-Melo, Zachary C. E. Hawley, Cristian A. Droppelmann, Michael J. Strong

**Affiliations:** ^1^Molecular Medicine Group, Robarts Research Institute, Schulich School of Medicine and Dentistry, Western University, London, ON, Canada; ^2^Department of Pathology, Schulich School of Medicine and Dentistry, Western University, London, ON, Canada; ^3^Department of Clinical Neurological Sciences, Schulich School of Medicine and Dentistry, Western University, London, ON, Canada

**Keywords:** stress granules, RNA, RNP, neurodegeneration, cancer, virus

## Abstract

Stress granules (SGs) are phase-separated, membraneless, cytoplasmic ribonucleoprotein (RNP) assemblies whose primary function is to promote cell survival by condensing translationally stalled mRNAs, ribosomal components, translation initiation factors, and RNA-binding proteins (RBPs). While the protein composition and the function of proteins in the compartmentalization and the dynamics of assembly and disassembly of SGs has been a matter of study for several years, the role of RNA in these structures had remained largely unknown. RNA species are, however, not passive members of RNA granules in that RNA by itself can form homo and heterotypic interactions with other RNA molecules leading to phase separation and nucleation of RNA granules. RNA can also function as molecular scaffolds recruiting multivalent RBPs and their interactors to form higher-order structures. With the development of SG purification techniques coupled to RNA-seq, the transcriptomic landscape of SGs is becoming increasingly understood, revealing the enormous potential of RNA to guide the assembly and disassembly of these transient organelles. SGs are not only formed under acute stress conditions but also in response to different diseases such as viral infections, cancer, and neurodegeneration. Importantly, these granules are increasingly being recognized as potential precursors of pathological aggregates in neurodegenerative diseases. In this review, we examine the current evidence in support of RNA playing a significant role in the formation of SGs and explore the concept of SGs as therapeutic targets.

## Introduction

Stress granules (SGs) have been described as a triage for mRNA during cellular stress where they either store translationally silent mRNA, transfer mRNA transcripts to processing bodies (p-bodies) where they will be degraded, or transfer mRNA back into polysomes for translation (Kedersha et al., 2000, 2002; Anderson and Kedersha, 2008). This SG–polysome–p-body axis has become well defined and, in doing so, has increased our understanding of SG function, composition, and dynamics and highlighted the essential role of SGs in mRNA metabolism and translational control during periods of stress to assist in cell response and recovery.

### Stress Granule Dynamics

Cellular stress can induce the phosphorylation of eukaryotic initiation factor 2 alpha (eIF2α), which is generally considered the trigger that induces SG assembly. However, SG formation can be independent of eIF2α phosphorylation and triggered by eukaryotic translation initiation factor 4A (eIF4A) (Low et al., 2005; Mazroui et al., 2006). In either case, the main goal of these signal transduction pathways is to release RNA molecules from polysomes to form SGs and inhibit translation (Kedersha et al., 2000; Buchan et al., 2011; Bounedjah et al., 2014). The release of RNA from polysomes allows RNA to act as a scaffold to nucleate RNA-binding proteins (RBPs) and initiate SG assembly (Bounedjah et al., 2014). This release of RNA from polysomes has led researchers to believe that RNA is translationally silent while in SGs. However, more recent data using single-molecule imaging of translating RNA has challenged this hypothesis showing that, while some mRNAs are translationally silent, there are a host of RNA molecules undergoing translation in SGs (Mateju et al., 2020), indicating that polysome release may not be the sole requirement for RNA recruitment into SGs.

The coalescence of RBPs and mRNA molecules into SGs creates a ribonucleoprotein (RNP) granule. This non-membrane bound organelle contains two subcompartments, the shell and the core. Cores are highly concentrated areas of mRNA and protein, which are surrounded by a less concentrated area, known as the shell, which is believed to be more dynamic (Jain et al., 2016). Little is known about the differences between these two subcompartments, although it is hypothesized that they differ in composition, function, and dynamics (reviewed in Protter and Parker, 2016).

The assembly of proteins during periods of stress results in the liquid–liquid phase separation (LLPS) of SGs that is primarily driven by weak electrostatic, hydrophobic, and homo- and heterotypic protein–protein interactions between RBPs that contain intrinsically disordered domains (Lin et al., 2015; Molliex et al., 2015; Murakami et al., 2015; Nott et al., 2015). While protein–protein interactions are critical for SG assembly, it has been shown that RNA can self-assemble and induce LLPS (Jain and Vale, 2017; Tauber et al., 2020a). Classically, it was thought that intrinsically disordered regions were the major drivers of LLPS through protein–protein interactions, but it has more recently become clear that RNA–RNA interactions are also playing an essential role.

These liquid-like structures are highly dynamic and continuously undergo adenosine triphosphate (ATP)-dependent remodeling (Jain et al., 2016). For example, recent work has shown that RNA-dependent DEAD box ATPases (DDX’s) are necessary for ATP hydrolysis in RNP granules, which allows for assembly, compartment turnover, and RNA release, and thus, providing cells spatial and temporal control of RNA processing (Hondele et al., 2019; Sachdev et al., 2019). Furthermore, once the stress abates, SGs start to disassemble in a process that is believed to be governed by several ATP-dependent mechanisms including inhibition of DNA/RNA helicases that stabilize SGs, activation of autophagic pathways via Valosin-containing protein (VCP) to promote protein degradation, or activation of protein chaperones such as heat shock proteins 40 and 70 (HSP40/70) (Protter and Parker, 2016). Therefore, ATP is critical for the rapid assembly, remodeling, and disassembly of SG components—important processes that are needed to avoid persistent granule formation, a phenomenon associated with disease states (Wolozin and Ivanov, 2019). Several lines of evidence have shown that RNA can mitigate excessive protein–protein interactions, which lead to pathological aggregates seen in several neurodegenerative disorders (Maharana et al., 2018; Mann et al., 2019; Zacco et al., 2019), highlighting the essential balance between protein–protein, RNA–protein, and RNA–RNA interactions needed for appropriate SG assembly and disassembly.

The overall rate of SG assembly has been shown to be dependent on the stress type. *In vitro* oxidative stress induced with sodium arsenite results in the formation of SGs within 30 min, while double-stranded DNA breaks induced by UV exposure *in vitro* results in a much slower formation of SGs spanning upward for 18 h (Kedersha et al., 2002; Moutaoufik et al., 2014). Sodium arsenite activates kinase heme-regulated inhibitor (HRI), which phosphorylates eIF2α allowing for rapid inhibition of translation and formation of SGs (Lu et al., 2001). However, under UV stress, SGs were observed to not coincide with global translation inhibition or eIF2α phosphorylation, but rather to coincide with S-phase arrest, which was hypothesized to prevent cell cycle progression until DNA damage was repaired (Moutaoufik et al., 2014). Therefore, the specificity of the stress response in UV-treated cells may explain this longer assembly of SGs, whereas sodium arsenite induces a general response, which is more rapid. Furthermore, in *in vivo* rodent models, an axonal crush can induce a stress response in dorsal root ganglion (DRG) within 3 h as SG-related proteins (i.e., G3BP1 and TIA-1) peak in expression (Moisse et al., 2009; Sahoo et al., 2018). After an ischemia–reperfusion injury, SGs were observed in ∼3 days within neurons located in the CA1 region of the hippocampus (Moisse et al., 2009; Ayuso et al., 2016). In contrast, pharmaceutical approaches (i.e., intragastric administration of sodium arsenite) can induce SG formation in the motor cortex within 2 h (Zhang et al., 2020). All these highlight that stress type has a major mechanistic impact on the formation of SGs both in *in vitro* and *in vivo* models.

Beyond these intrinsic dynamics of SGs, SGs also closely interact with other RNP granules to regulate RNA expression and metabolism (reviewed in Riggs et al., 2020). As previously discussed, SGs are in a dynamic equilibrium with polysomes. This dynamic equilibrium between SGs and polysomes has been shown through pharmaceutical approaches *in vitro*, where exposure to cycloheximide (a polysome stabilizer) or puromycin (a polysome destabilizer) results in decreased or increased SG formation, respectively (Kedersha et al., 2000; Riggs et al., 2020). Furthermore, SGs also closely interact with another RNP granule known as a p-body to regulate mRNA degradation (Kedersha et al., 2005; Wilczynska et al., 2005; Aulas et al., 2015). SGs and p-bodies rapidly and continuously exchange both RNA and protein molecules. While the exchange rate of protein between SGs and p-bodies ranges from seconds to minutes, the exchange rate of RNA molecules is less certain (Andrei et al., 2005; Kedersha et al., 2005; Mollet et al., 2008). The half-life of a single mRNA molecule is ∼1 min in an SG before it is shuttled out, suggesting that RNA is briefly stored in SGs before it either goes into p-bodies or re-enters polysomes to be degraded or translated, respectively (Mollet et al., 2008).

### Stress Granule Composition

T-cell-restricted intracellular antigen-1 (TIA-1) and Ras-GTPase-activating protein SH3-domain-binding protein 1 (G3BP1) are two RBPs that are necessary components for SG formation (Tourriere et al., 2003; Gilks et al., 2004). The aggregation of TIA-1 and G3BP1 into cytoplasmic SGs are believed to be nucleating factors by interacting with free RNA allowing for downstream SG assembly (Anderson and Kedersha, 2008). G3BP1 differs from TIA-1 in that it interacts with 40S ribosomal subunits during stress, whereas SG nucleating factor TIA-1 acts to coalesce specific RNA transcripts (Piecyk et al., 2000; Kedersha et al., 2016). Furthermore, G3BP1 has been shown to act as a molecular switch needed to trigger RNA-dependent LLPS of stress granules by sequestering free RNA to generate RNA–protein condensates (Guillen-Boixet et al., 2020; Yang et al., 2020). However, U2OS cells lacking G3BP1 and G3BP2 are still able to form stress granules under osmotic or heat stress, but not under oxidative or endoplasmic reticulum (ER) stress (Kedersha et al., 2016; Yang et al., 2020) indicating that G3BP proteins are necessary for nucleation of SGs under specific stress conditions and not others.

Once nucleation occurs, this allows for the recruitment of other proteins with a variety of functions, creating a complex composition that allows SGs to dynamically exchange RNA and protein molecules. These additional proteins associated with SG include translation initiation factors, RBPs, kinases, phosphatases, ATPases, guanosine triphosphatases (GTPases), methyltransferases, ribosyltransferases, glucosyltransferases, DNA/RNA helicases, and ubiquitin-modification enzymes (Jain et al., 2016; Markmiller et al., 2018; Kuechler et al., 2020). The specific protein and RNA composition of SGs has been shown to be dependent on the stress, length of stress, cell type, and cellular localization (Buchan et al., 2011; Protter and Parker, 2016; Markmiller et al., 2018; Reineke et al., 2018; Reineke and Neilson, 2019; Kuechler et al., 2020). Despite the diverse SG proteome, 78–95% of SG composition is RNA (Khong et al., 2017).

During periods of oxidative stress, mitochondrial-based transcripts have been shown to be highly enriched with G3BP1-positive SGs, whereas cytoprotective genes were polysome enriched (Somasekharan et al., 2020). This indicated that during oxidative stress, cells translationally silenced genes that reduced mitochondrial activity to prevent further oxidation and promote the expression of genes needed to return to a homeostatic state. After an axonal injury in rodent DRG neurons, G3BP1 forms SG-like structures within the axon, which are increasingly more abundant in more distal regions, closer to where the injury occurs. Furthermore, G3BP1 sequesters *Importin β1* (*Impβ1*) mRNA, but dissociates *Neuritin 1* (*Nrn1*) allowing for decreased and increased translation of these two transcripts, respectively, which is believed to promote rapid regenerative processes (Sahoo et al., 2018). This highlights the role of SGs and G3BP1 in regulating genes not only involved in stress-specific needs but as well as compartmental needs (i.e., the axon of a neuron) during periods of stress.

Stress granules can be further defined as being canonical or non-canonical depending on their protein composition. Canonical SGs tend to contain pro-apoptotic factors to suppress their function and prevent apoptosis, while non-canonical SGs lack these apoptotic factors indicating that programmed death may be activated in these cells (Fujimura et al., 2012; Aulas et al., 2018; Reineke et al., 2018; Reineke and Neilson, 2019). Typically, canonical SGs include ribosomal S6 kinase 2 (RSK2), histone deacetylase 6 (HDAC6), importin α1, rho-associated coiled-coil containing protein kinase 1 (ROCK1), TNF receptor-associated factor 1 (TRAF2), JUN N-terminal kinase (JNK), mitogen-activated protein kinase 7 (MKK7), Ras homolog family member A (RhoA), WD repeat domain 62 (WDR62), and receptor of activated protein C kinase 1 (RACK1). The recruitment of these proteins prevents signal cascades that result in apoptotic signaling (Kim et al., 2005; Kwon et al., 2007; Eisinger-Mathason et al., 2008; Fujimura et al., 2010, 2012; Tsai and Wei, 2010; Wasserman et al., 2010).

The formation of either canonical or non-canonical SGs can depend on both the length and type of stress. For example, the major difference in SG composition between an acute oxidative stress induced by sodium arsenite and a chronic nutrient starvation stress is the exclusion of RACK1 from chronic SGs (Reineke et al., 2018). This is significant because RACK1 recruitment to SGs is essential for inhibition of the mitogen-activated protein kinase (MAPK) pathway, preventing cell apoptosis and promoting cell survival (Arimoto et al., 2008; Reineke et al., 2018; Park et al., 2020). However, it is unclear whether this change in composition is due to stress duration as suggested, or whether it is due to differences in stress type, as specific stresses can also induce non-canonical SGs. Selenite-induced stress drives the formation of non-canonical SGs, resulting in the exclusion of RACK1, as well as other canonical SG components including HDAC6 and Importin α1 (Fujimura et al., 2012). Furthermore, nitric oxide-induced SGs, which do include RACK1, exclude eukaryotic translation initiation factor 3B (eIF3B), which is found in canonical SGs. This exclusion is believed to drive apoptotic signaling induced by nitric oxide stress (Aulas et al., 2018). Therefore, SGs can function as hubs to regulate apoptotic signaling by sequestering specific cell-signaling components, like RACK1, which is dependent on both stress type and, potentially, duration (Kedersha et al., 2013; Reineke and Neilson, 2019).

This knowledge of the breadth of SG protein composition has allowed researchers to characterize a major difference between SG subtypes based on differences in stress, stress duration, and canonical versus non-canonical. However, understanding that the majority of SG composition is RNA has opened up new avenues in understanding the genesis of these SG subtypes.

### Stress Granule Compartmentalization

Stress granules are generally found within the cytoplasm of the cell, but it has become increasingly recognized that the specific compartmentalization of SGs in the cytoplasm may play a critical role in the stress response. For example, the compartmentalization of SGs to different regions of a neuron (i.e., soma, dendrite, axon, or synapse) is important for functions related to synaptic plasticity or axonal regeneration (Vessey et al., 2006; Martin et al., 2013; Sahoo et al., 2018). In yeast, nutrient deprivation results in SG formation at the plasma membrane, which is dependent on eisosomes—subcortical membrane structures that mark sites of endocytosis. Localization of SGs at the plasma membrane via interactions with eisosomes allows for sequestering of active protein kinase C-like 1 (Pkc1) into SGs, which potentiates cell recovery (Amen and Kaganovich, 2020). Differential compartmentalization of SGs to different cytoplasmic regions appears to be a critical aspect of cell recovery during stress by assisting with localized needs.

Nuclear SGs, termed nuclear stress bodies (nSB), have also been described as a component of the heat shock response. The main component of the nSB is the transcription factor heat shock factor 1 (HSF1), which nucleates in the SAT III region of DNA and transcribes a series of non-coding RNAs (Biamonti and Vourc’h, 2010). TAR DNA-binding protein 43 (TDP-43) has also been observed to form nSBs in response to oxidative and ER stress. These nSBs were observed to have a similar shell-core architecture as cytoplasmic SGs. TDP-43-positive nSBs are responsible for alleviating cell toxicity during periods of stress, but the mechanism that drives this cytoprotective affect is unclear (Malik and Barmada, 2020; Wang C. et al., 2020).

Stress granules are critical determinates for RNA-mediated gene expression in which the composition of these molecules is driven by specific cell stresses. They can act as an intermediate between polysomes and p-bodies to regulate RNA translation and degradation, respectively. Overall, this regulation of mRNA metabolism/expression by SGs allows cells to maintain homeostasis in response to a stress, or when the stress cannot be overcome, induce apoptotic pathways. In recent years, RNA has become increasingly implicated in SG assembly and disassembly. Studying RNA composition of SGs and its function is of outmost importance not only because RNA is the major component of SGs but also because as we are going to see later, RNA itself is able to phase separate in the absence of proteins and to function as efficient scaffolds for protein complexes. The more we understand about RNA molecules in SGs, the more we are going to increase our knowledge about SG dynamics, and the closer we are going to be to finely regulate these transient organelles and to develop therapeutic tools for different types of diseases.

## RNA in the Formation and Function of Stress Granules

Several RBPs have been observed to drive the formation and dynamics of SGs. Overall, protein drivers contain intrinsically disordered regions (IDRs) and/or govern low-affinity multivalent interactions (Kato et al., 2012; Li et al., 2012; Banani et al., 2017; Van Leeuwen and Rabouille, 2019). The function of RNA species in this process is less clear but has recently begun to be uncovered (Van Treeck and Parker, 2018; Van Treeck et al., 2018). There is now substantial evidence that RNA molecules are not just casual passengers in the formation of RNP granules, but drivers of SG formation, determining through RNA–RNA and RNA–protein interactions, at least in part, granule composition.

### The Dynamic Nature of RNA

**FIGURE 1:**
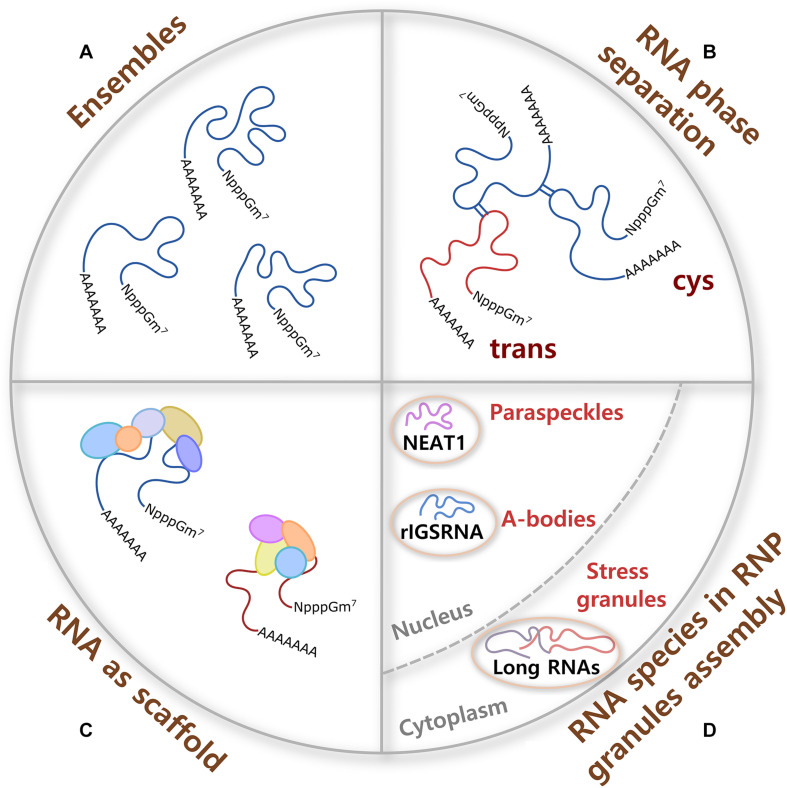
RNA contributions to RNP granule formation. **(A)** A single molecule of RNA can adopt different conformations called ensembles, some of them formed with high probability and others not, which provide flexibility to form multivalent interactions and the potential to have different molecular functions. **(B)** *Cis* and *trans* RNA–RNA interactions participate in RNA phase separation in the absence of proteins. RNA interactions are specific or promiscuous, and are modulated by ionic strength, osmolarity, pH, temperature, and crowdedness. **(C)** Because of their large size, RNA molecules are better scaffolds for multiprotein complex formation than proteins. RNA–protein interactions are formed through negatively charged phosphate groups of RNA and different RNA-binding proteins (RBPs). **(D)** Certain ribonucleoprotein (RNP) granules require specific RNA species for their assembly. In the nucleus, non-coding RNAs nuclear paraspeckle assembly transcript 1 (NEAT1) and RNA derived from the rDNA intergenic spacer (rIGSRNA) are necessary for the formation of paraspeckles and A-bodies, respectively. In the cytoplasm, even though no specific RNA molecule is required for stress granule (SG) assembly, long coding and non-coding RNAs form extended RNA–RNA networks that are necessary for SG formation.

RNA is a highly flexible and dynamic molecule whose folding is more promiscuous than protein folding due to the simpler alphabet and base-pairing rules. An RNA molecule spontaneously adopts a vast number of conformations that together are called ensembles (Figure 1A), some of which may form with high probability, while others may be very rare (Ganser et al., 2019). These conformers could have completely different functions. For example, certain RNA viruses such as human immunodeficiency virus 1 (HIV-1) can switch between different RNA secondary structures to execute different functions required for different steps of the viral replication cycle. The 5′UTR of the HIV-1 genome can form two secondary conformations, a branched multiple hairpin structure involved in dimerization and packaging and a long-distance interaction conformation that participates in transcription and translation (Abbink et al., 2005). In general, local and global structural rearrangements are common in RNA molecules and are critical for its functions. Importantly, RNA has the ability to change structure in response to molecular effectors and environmental cues. Cellular modifiers such as metabolites, ions, and RBPs change the abundance of one or two pre-existing conformations of the RNA ensemble (Ganser et al., 2019).

Homo (*cis*) and heterotypic (*trans*) RNA–RNA (Figure 1B) and RNA–protein interactions participate in the assembly of RNP granules. RNA–RNA interactions can be specific or promiscuous and include Watson–Crick, non-Watson–Crick, base stacking interactions, and tertiary interactions such as purine minor grove interactions, ribose zippers, and tetraloop–tetraloop receptor interactions. Importantly, aromatic interactions (π–π) dictate the shape of most RNA tertiary and high-order structures. Tertiary and quaternary interactions can be stabilized by Mg^+2^ ions and, to a lesser extent, by K+. In addition, RNA interactions are modulated by several other parameters such as ionic strength, osmolarity, pH, temperature, and crowdedness (Draper, 2004; Bou-Nader and Zhang, 2020). Ultimately, multivalent RNA–RNA interactions can induce RNA oligomerization, condensation, and phase separation.

Along with this, the ability of a single long RNA molecule to interact through negatively charged phosphate groups with different RBPs, which in turn form multiprotein complexes (Figure 1C), make RNA ideal molecules to command the formation and composition of RNA assemblies. Moreover, it has been shown that the RNA structure drives interaction with proteins and that a highly structured RNA can rearrange the composition of a protein aggregate (Sanchez De Groot et al., 2019). Interestingly, even though long mRNAs show high partition into SGs (see below), there is only a modest enrichment of SG-resident proteins on SG-enriched mRNAs. This observation suggests that mRNA length contributes to SG assembly through RNA–RNA interactions (Khong et al., 2017). Then, sequence, length, structure, and chemical modifications of translationally silent-RNAs can potentially determine and regulate a large spectrum of interactions that every RNA molecule establishes in RNP assemblies.

**FIGURE 2:**
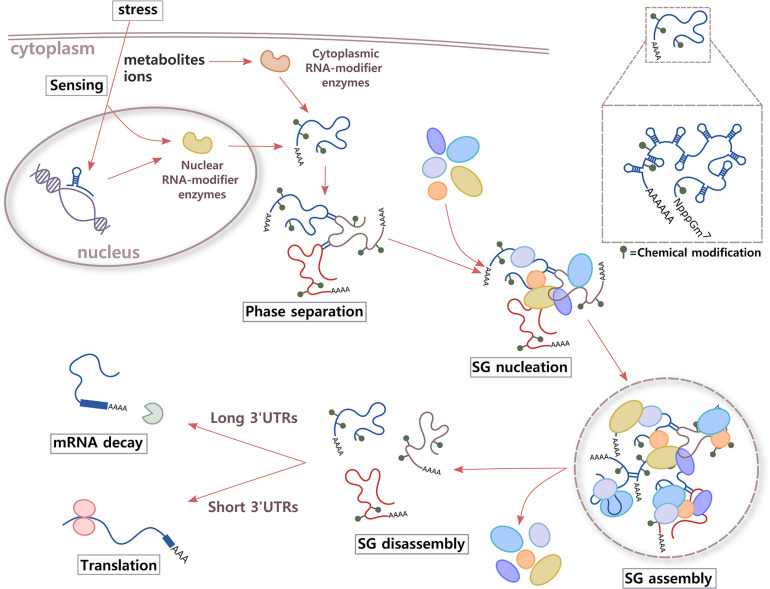
Proposed model. Immediately after integrate stress response (ISR) signaling is activated in the cell, RNA might participate in sensing changes in metabolites and/or ions of the environment through two different mechanisms. Aptamer domains of RNAs that are being transcribed and enzymes that add or erase RNA chemical modifications could bind small molecules whose levels increase inside the cell under stress conditions. As a result, alterations in structure and local charges in RNA in crowded environments of translationally stalled mRNAs could contribute to increase *cis* and *trans* RNA–RNA interactions and phase separation. RNA molecules could also work as highly efficient scaffolds for multiprotein complexes through RNA–protein interactions, leading to SG nucleation and assembly. After the stress stimulus disappears, and metabolites and ions return to basal levels, RNA conformation rearrangements could trigger SG disassembly, releasing RBPs and mRNAs. Then, RBPs are re-compartmentalized inside the cell to sites where they exert different functions, and mRNAs are destined to translation (short 3′UTRs) or degradation (long 3′UTRs).

In response to stress, eukaryotic cells activate a common signaling pathway called the integrated stress response (ISR). The stress sensors are kinases HRI (heme-regulated inhibitor kinase or EIF2AK1, eukaryotic translation initiation factor 2-alpha kinase 1; activated by different stressors in addition to heme deficiency), PKR (dsRNA-activated protein kinase or EIF2AK2; activated by UV exposure, viral infections, and heat shock, among others), PERK (PKR-like endoplasmic reticulum kinase or EIF2AK3; activated during ER stress), and GCN2 (general control nonderepressible 2 or EIF2AK4; activated by different stressors such as amino acid deprivation); all of which phosphorylate eIF2α (Wek et al., 2006; Donnelly et al., 2013; Krishna and Kumar, 2018). This event causes a reduction in global protein synthesis and triggers the assembly of SGs (Pakos-Zebrucka et al., 2016). The next steps in SG assembly are less well characterized. For instance, after ISR activation and translational arrest, is the high concentration of untranslating RNAs the only trigger of phase separation? Could a signal that is rapidly transmitted inside the cell also contribute to initiate RNA assembly and nucleation of SGs? One possibility is that metabolites and ions activate enzymes involved in RNA chemical modification, changing the RNA structure to favor RNA–RNA interactions (see below). Another interesting idea is that metabolite and ion alterations could be sensed by the RNA molecule itself (Figure 2). The small size, ease of movement within the cell, and fast response of metabolites and ions when needed make them excellent molecules to communicate environmental changes inside the cell. Zn^2+^, for example, promotes rapid multimerization, phase separation, and subsequent localization of TIA-1 into SGs (Rayman et al., 2018). For many years, the binding of small molecules was thought to be exclusive to proteins. It is now accepted that RNA species can also bind metabolites and act, for example, as riboswitches, highly conserved structural elements, or aptamers that bind intracellular ligands with high affinity and selectivity, and regulate transcription, splicing, and translation of mRNAs.

Metabolites such as nucleotides, amino acids, and coenzymes, and ions like Mg^2+^ and H^+^ do not have the chaperone activity necessary to drive secondary structure transitions over the associated large-energy barriers. This is the reason that they act to direct RNA to different folding pathways during RNA co-transcriptional folding (Dethoff et al., 2012). The aptamer domain is the first part of the RNA to be transcribed and folded into a shape capable of ligand binding. A short pause right after provides the aptamer domain time to “interrogate” the cellular environment for the presence of ligand (Wickiser et al., 2005). Natural riboswitches exist across all domains of life and are increasingly found in eukaryotes (Lotz and Suess, 2018).

Besides triggering regulation of gene expression, the binding of a metabolite or ion could potentially modify the properties and functions of RNA molecules in other manners. Conformational changes of the RNA at the secondary, tertiary, and quaternary structural levels, could make it transiently relocate inside the cell, while the stimulus is present, and increase its interactions with other RNAs or proteins to initiate phase separation and the formation of different types of RNA granules (Figure 2).

### RNA Induces Phase Transitions

RNA molecules tend to assemble *in vitro* and in cells whenever there is a high local concentration of RNA molecules through a process called LLPS (Figure 1B). RNA can drive phase separation, regulate the physical properties of droplets, and control the identity of liquid compartments independent of protein–protein interactions (Lin et al., 2015; Zhang et al., 2015; Jain and Vale, 2017; Langdon et al., 2018; Garcia-Jove Navarro et al., 2019). Moreover, RNA usually needs a lower concentration to condense *in vitro* than intrinsically disordered proteins (Van Treeck and Parker, 2018).

Important information regarding the role of RNA molecules in the assembly of RNA granules has emerged from germ granules of *D. melanogaster*, *C. elegans*, *and D. rerio*. Germ granules, formed only in germ cells, are a specific type of RNP assembly that promotes segregation of mRNAs with opposing regulatory needs and co-regulation of mRNAs from the same biological process (Buchan, 2014). A recent study with germ granules has determined that multiple mRNAs derived from the same gene self-assemble into homotypic clusters in a sequence-independent manner. mRNA localization into germ granules is governed by specific RNA regions, while mRNA self-assembly does not involve a specific sequence but the whole mRNA (Trcek et al., 2020). Glycolytic (G) bodies, another type of RNA granule that assembles glycolytic enzymes in the cytoplasm under stress conditions, are also nucleated and maintained in their structural integrity by RNA, showing the broad importance of RNA in the formation of different RNP granules (Fuller et al., 2020).

Multivalent intermolecular base pairings are also created by GC repeats that are involved in many repeat expansion diseases such as CAG in Huntington disease and spinocerebellar ataxias and a hexanucleotide expansion GGGGCC (G_4_C_2_) in the chromosome 9 open reading frame 72 (*C9orf72*) gene in amyotrophic lateral sclerosis (ALS) and frontotemporal dementia (FTD). It has been shown that GC repeats in a G-quadruplex conformation originate in RNA sol–gel transitions *in vitro* and in cells, without requiring proteins, in a repeat length and structure-dependent manner and at a similar repeat number as in nucleotide repeat expansion disorders. RNA foci are formed by phase separation of the repeat-containing RNA in cells and can be dissolved by agents that disrupt RNA gelation (Fay et al., 2017; Jain and Vale, 2017). However, G_4_C_2_ RNA repeats only recruit a subset of SG proteins to foci, suggesting that multiple different RNA molecules might be necessary to fully recapitulate SG composition (Fay et al., 2017). RNA gelation explains why repeat expansion diseases appear to be triggered after a threshold number of nucleotide repeats and why distinct repeat expansions in different genes can result in similar clinical phenotypes (Jain and Vale, 2017).

Importantly, the first evidence of the importance of RNA in the formation of RNP granules comes from studies using the pharmacological inhibition of translation. It has been shown that cycloheximide, an elongation inhibitor, stabilizes polysomes and prevents assembly of SGs and P-bodies. On the contrary, puromycin, a tRNA analog that induces premature termination, destabilizes polysomes and enhances SG formation. Experiments have demonstrated that SGs and P-bodies are in a dynamic equilibrium with polysomes and that mRNAs released from polysomes are necessary for SG assembly (Kedersha et al., 2000; Andrei et al., 2005; Riggs et al., 2020). Studies in yeast have shown that mixtures of cellular RNAs form assemblies *in vitro* that phase separate. RNA itself is enough to trigger yeast SG formation in a process that is ATP sensitive; however, transcript variety is necessary to form a canonical SG. Under physiologically relevant conditions, RNAs enriched in assemblies from total yeast RNA recapitulates the SG transcriptome *in vivo.* These observations support the idea that RNA–RNA interactions contribute to both formation and RNA composition of SGs (Van Treeck et al., 2018; Begovich and Wilhelm, 2020).

RNA structure is emerging as a critical regulator of phase separation. mRNA secondary structure establishes the specificity of phase separation through self-association of RNAs, ultimately determining whether the RNA is recruited or excluded from liquid compartments (Figure 1B) (Langdon et al., 2018). RNA helicases that unwind secondary structures and RNA–RNA interactions limit RNA condensation under physiological conditions *in vitro* and SG formation in cells (Tauber et al., 2020b). Interestingly, it was shown that RNA also induces structural changes in proteins critical in SG formation. Under non-stress conditions, G3BP1, well-known to be necessary for SG assembly, adopt an auto-inhibited compact state stabilized by intramolecular electrostatic interactions between the positively charged RG-rich region and a disordered acidic region. In stress conditions, RNA is released from the polysomes and competes with G3BP1 auto-inhibitory interactions to liberate the RG-rich region, triggering a conformational change that favors G3BP1 clustering through RNA–protein interactions. This results in RNA–G3BP1 condensates of low protein density that recruit additional proteins to promote SG maturation (Guillen-Boixet et al., 2020).

Recently, it has been proposed that since RNA condensation happens spontaneously, RBPs that are associated to RNP granules could be, in fact, RNA chaperones that regulate the condensation process both kinetically and thermodynamically. Interestingly, Tauber et al. (2020b) have classified all the SG proteins into five groups: (a) kinetic RNA condensers, proteins that increase the rate of *trans* RNA–RNA interaction formation; (b) thermodynamic RNA condensers, proteins that reduce ΔG of RNP granulation through RNA binding; (c) kinetic RNA decondensers, proteins that reduce activation energy barriers and decrease the valency of RNAs to promote the dissociation of *cis* or *trans* RNA interactions and accelerate RNA refolding; (d) thermodynamic RNA decondensers proteins that bind RNA with high affinity to restrict the sites or conformations that are available for *trans* RNA–RNA interactions; and (e) client/unknown proteins. According to this model, most SG proteins would assist to control RNA condensation through ATP-dependent processes (Tauber et al., 2020a).

### RNA Is Critical for Ribonucleoprotein Granule Assembly

Certain RNA species are essential for the formation of RNA granules upon stress, functioning as scaffolds for protein complex formation (Figures 1C, 2). For example, Whi3 is an RBP with a polyQ-expansion that is essential for the special patterning of cyclin (*CLN3*) and formin (*BNI1*) transcripts in the cytosol of large cells in fungi (Lee et al., 2013, 2015). Different mRNAs that are physiological targets of Whi3 drive Whi3 assembly into dynamic liquid-like droplets with distinct properties that readily fuse with one another upon contact. Over time, Whi3 droplets mature forming fibrillar structures (Zhang et al., 2015).

Another example is the seeding of nuclear amyloid bodies (Figure 1D, A-bodies), inducible membraneless nuclear compartments formed by RNA and heterogenous proteins that adopt an amyloid-like state and work in detention of protein and RNA species. Under stress, clusters of long low-complexity dinucleotide repeats (CU or AG) of a non-coding RNA derived from the rDNA intergenic spacer (rIGSRNA) accumulate in the nucleolus, where they facilitate charge-based interactions with short cationic peptides to induce liquid-like foci. Then, accumulation of proteins with fibrillation propensity in these RNA foci activates A-body biogenesis (Wang M. et al., 2018).

Perhaps the most well-known example of RNAs as nucleating factors of RNP granules is the long non-coding RNA nuclear paraspeckle assembly transcript 1 (NEAT1) in the formation of stress-inducible nuclear bodies called paraspeckles (Figure 1D). There are two proposed functions for paraspeckles: as nuclear retention of inverted repeats-containing mRNAs and as a molecular sponge for RBPs (Nakagawa et al., 2018). It has been shown that NEAT1 has an essential architectural role for paraspeckles formation, working as a scaffold for *Drosophila* behavior human splicing (DBHS) proteins that include paraspeckle component 1 (PSPC1), non-POU domain-containing octamer-binding protein (P54NRB/NONO), and splicing factor proline and glutamine rich (SFPQ/PSF). NEAT1, unlike DBHS proteins that are also necessary for paraspeckle formation, is a limiting factor in the formation of these granules. The two transcript isoforms of NEAT1, NEAT1_1 (short, exclusively localized in paraspeckles) and 1_2 (long, also found in the nucleoplasm) are necessary to maintain paraspeckle integrity (Chen and Carmichael, 2009; Clemson et al., 2009; Sunwoo et al., 2009; Souquere et al., 2010; Chujo et al., 2017).

No master scaffolding RNA species have been found in cytoplasmic RNA granules. However, cytoplasmic untranslated mRNAs of long coding regions and 3′ untranslated region (UTR) lengths form extended RNA–RNA networks *in vivo* and are necessary for the assembly of SGs (Figure 1D) (Khong et al., 2017). It has been observed that upon polysome dissociation, the delivery of free exogenous mRNA creates a high-local concentration of RNA that triggers SG assembly and works as scaffolds for protein multimerization (Figure 1C). Conversely, the increase in RBPs can prevent aggregation of mRNA by forming isolated mRNPs. Moreover, experiments in enucleated cells have shown that shuttling of RBP from the nucleus to the cytoplasm seems not to be necessary for SG formation (Bounedjah et al., 2012). Using a yeast cytoplasmic extract system for SG reconstitution, it has been observed that RNA composition of the condensate, ATP level, and ATPase activity regulate SG formation (Begovich and Wilhelm, 2020). In cells, depletion of ATP promotes condensation into SGs in the absence of protein factors required for SG formation, suggesting that cells use ATP to limit SG formation (Tauber et al., 2020a). ATP dependence is in agreement with the observation that *in vivo* assembly and disassembly of SGs is regulated by chaperones and/or helicases (Walters et al., 2015; Jain et al., 2016; Hondele et al., 2019; Tauber et al., 2020b).

Interesting studies using single-cell tracking have allowed us to observe the movement of single mRNAs in and out SGs and P-bodies. These experiments have shown that mRNAs move bidirectionally between these two types of granules. They have also showed that non-translating mRNAs form stable and sometimes rigid associations within SG granules. The stability in these interactions increases with mRNA length and granule size. In addition, live cell imaging has demonstrated that mRNAs can extend beyond the protein limits in the granule, possibly participating in interactions with other types of granules and the transport machinery (Moon et al., 2019).

## Rna Species in Stress Granules

The role of proteins in the formation of RNP granules has been a matter of study for many years. More recently, advances in the study of RNAs in the dynamics of formation of membraneless organelles are helping to create a more complete picture of the sequence of events in the assembly and disassembly of these transient structures. Additionally, the development of SG purification techniques combined with transcriptomics has begun to elucidate the RNA composition of SGs and the role of RNA chemical modifications in the regulation of SG formation.

### Transcriptomics of Stress Granules

During cellular stress, the sequestration of translational-suppressed mRNAs into SGs is associated with enhanced cell viability (Lavut and Raveh, 2012). Recently, the characteristics and identities of these RNA molecules have begun to be uncovered. Different techniques have been used to elucidate the transcriptomes of RNP granules. In general, they are based in differential centrifugation alone or together with fluorescence-activated particle sorting or immunopurification (Hubstenberger et al., 2017; Khong et al., 2017, 2018; Namkoong et al., 2018).

Analysis of the SG transcriptome has shown that only 10% of bulk mRNA molecules accumulate in mammalian SGs; however, proportionally, more RNAs than proteins are present in SGs compared with the cytoplasm (Bounedjah et al., 2014; Khong et al., 2017). Thousands of different mRNA species can localize into SGs. These SG mRNAs are similar under different stresses and have less ribosome density, indicating that inefficiently translated RNAs preferably accumulate into SGs. Remarkably, mammalian SG cores concentrate a specific type of mRNAs. SG-enriched mRNAs are long (average length 7.1 kb); the length of coding regions and UTRs of mRNAs correlate with accumulation in SGs. Mammalian and yeast long non-coding RNAs (lncRNAs) also concentrate into SGs (average length 1.9 kb), demonstrating that length has an important role in targeting both coding and noncoding transcripts to SGs. This is crucial for the dynamics of SG formation because long transcripts can have more conformational stages and engage in more RNA–RNA and RNA–protein interactions. In addition, the existence of lncRNAs in SGs demonstrates that prior translation *per se* is not a requirement for RNA accumulation into SGs (Khong et al., 2017; Van Treeck et al., 2018). Others have also observed that different types of stresses such as heat shock and oxidative stress induce a conserved pattern of RNP granule targeting. Only a small subset of translationally suppressed mRNAs from survival and proliferation genes, characterized by extended lengths and AU-rich elements (AREs), are enriched in RNP granules (Namkoong et al., 2018). More recently, Somasekharan et al. (2020) reported on G3BP1-associated transcripts using APEX-based proximity tagging and compared the data with the studies from Namkoong et al. (2018) and Khong et al. (2017), finding 2% and 38% of similarity, respectively (Khong et al., 2017; Namkoong et al., 2018; Somasekharan et al., 2020). Different cell lines, arsenite incubation times and concentration, and different methodologies to isolate SGs might explain the different results obtained.

Analysis of newly made RNAs after stress has revealed that cells use alternative polyadenylation (APA) sites as a mechanism of both SG assembly and mRNA stability control (Figure 2). Long 3′UTR variants bind TIA-1 more efficiently via U-rich motifs, associating with SGs, and later, they are destined to degradation. In contrast, short 3′UTRs evade RNA clearance and maintain mRNA levels after stress (Zheng et al., 2018). Interestingly, it has been shown that for G3BP1 binding, CDS length seems to be more important than 3′UTR length (Khong et al., 2017).

A comparison of the different RNP granule isolation techniques performed by Matheny et al. (2019) demonstrated that while centrifugation methodologies provide an approximation of the SG transcriptome, a more accurate determination is obtained by immunopurification of SGs. Using this technique, they observed that transcripts that are strongly enriched within P-bodies tend to have higher levels of AU composition, while transcripts that are strongly enriched within SGs tend to have higher levels of GC content. Overall, they determined that the RNA compositions of SGs and P-bodies are surprisingly similar under oxidative stress. This suggests that specific characteristics of transcripts from the same gene such as 3′UTR variants, lengths of poly(A)-tails, and RNA modifications determine their association to SGs or P-bodies (Matheny et al., 2019). In fact, it has been shown that the major determinant of mRNA enrichment to germ granules in *D. melanogaster* is the 3′UTR (Rangan et al., 2009).

Using tail-end displacement sequencing (TED-Seq) for transcriptome-wide profiling of poly(A) lengths, Woo et al. (2018) observed that mRNAs in the whole pellet of ER stress-induced RNA granules have shorter poly(A) tails than in the cytoplasm. This might be a result of the recruitment of short-tailed mRNAs to the granules and/or the active de-adenylation inside the granules. Whether shortening of poly(A)-tails observed in RNA granules happens in different stress conditions and in SGs and/or P-bodies, is an important question that requires further investigation (Woo et al., 2018).

### RNA Modifications in Stress Granule Formation

Cumulative evidence has shown that the cellular response to stress is finely regulated through post-translational modification (PTM) of proteins. The specific function of PTMs in SG dynamics has been extensively studied, leading to the proposal that alterations in physicochemical properties of modified amino acids regulate SGs, weakening or enhancing the multivalent interactions between macromolecules, or recruiting or excluding macromolecules from the granules (Hofweber and Dormann, 2019; Wang F. et al., 2020).

Recently, the role of RNA chemical modifications in SG formation dynamics has started to be revealed. RNA modifications are critical at different steps of the RNA metabolism, from transcription and splicing, to stability, translation, and RNA function. The mRNA epitranscriptome contains methyl, hydroxymethyl, acetyl, formyl, as well as the nucleoside isomers, pseudouridine and inosine. RNA molecules undergo a variety of reversible and irreversible modifications through a highly regulated process in which different enzymes participate. Writer enzymes transfer specific chemical groups to the RNA molecule, eraser enzymes remove them, and reader enzymes recognize modified nucleotides (Jones et al., 2020).

The most prevalent mRNA modification is N6-adenosine methylation (m^6^A). This modification has been found in tRNAs and rRNAs and is enriched around the stop codon of mRNAs and in 3′ UTRs. Its role is to regulate mRNA stability, localization, splicing, and translation (Dominissini et al., 2012; Meyer et al., 2012; Meyer and Jaffrey, 2014; Wang et al., 2014; Zhou et al., 2015; Huang and Yin, 2018). m^6^A tag readers YTH domain-containing proteins (YTHDF1, YTHDF1-3) and insulin-like growth factor 2 mRNA-binding proteins (IGF2BP1-3) mediate methylation effects (Nachtergaele and He, 2018). However, two other readers, fragile X mental retardation protein (FMR1) and leucine-rich pentatricopeptide repeat containing (LRPPRC) also have been associated with this modification (Arguello et al., 2017).

A less common mRNA modification is methylation of N1-adenine (m^1^A). This highly conserved modification not only adds a methyl group but also a positive charge to the RNA at the Watson–Crick interface, blocking RNA base pairing. Importantly, m^1^A increases in mRNAs in response to stress. m^1^A is also a well-known modification in rRNAs and tRNAs, playing roles in maintaining their biological functions. Also, it has been shown to be enriched around the start codon of mammalian transcripts where it promotes translation in methylated mRNAs and has been found in certain lncRNAs (Dominissini et al., 2016; Xiong et al., 2018).

Chemical modifications that alter architecture and charge in RNA molecules could affect the dynamics of RNP granule assembly and disassembly by modifying RNA, RNA–RNA, and RNA–protein interactions (Figure 2). One of the first evidences of the participation of RNA modifications in the dynamics of SGs was the observation that m^6^A disrupts RNA binding by G3BP1/2, ubiquitin-specific peptidase 10 (USP10), cell cycle-associated protein 1 (CAPRIN1), and RNA-binding motif protein 42 (RBM42), all proteins of SGs (Arguello et al., 2017; Edupuganti et al., 2017). Later, high-throughput RNA sequencing techniques and isolation of RNA granules are helping to understand the specific role of RNA modifications in SG dynamics. Isolation of SG mRNAs using photo-activatable ribonucleoside cross-linking and immunoprecipitation (PAR-CLIP) has shown that under oxidative stress, more than 50% of the SG transcripts contain m^6^A modifications. A higher proportion of methylation sites and a higher number of methylation sites per transcript was observed under stress condition. The analysis of the distribution of oxidative stress-induced methylation showed that they predominantly localize in the 5′UTRs and 5′ vicinity of CDSs. Interestingly, these modifications provide a selective mechanism for mRNA triage from the translatable pool to SGs, mediated by YTHDF3 (Anders et al., 2018). Regarding the mechanism of RNP granule formation, *in vitro* studies have shown that m^6^A-binding proteins YTHDF1, YTHDF2, and YTHDF3 undergo LLPS. This phase separation is markedly enhanced by mRNAs that contain multiple m^6^A residues, acting as multivalent scaffolds for the binding of YTHDF proteins (Ries et al., 2019).

More recently, Fu and Zhuang (2020) confirmed the enrichment of m^6^A- and m^1^A-modified mRNAs in SGs. They also demonstrated that YTHDF proteins are crucial for SG formation. When they depleted reader enzymes YTHDF1/3, enrichment of methylated and unmethylated mRNAs in SGs was prevented, and SG formation was inhibited. Both the N-terminal intrinsically disordered domain and the C-terminal m^6^A-binding YTH domain of YTHDF showed to be crucial for SG formation (Fu and Zhuang, 2020). Recently, m^1^A-modifications of RNA have also been involved with the dynamics of SG formation. Both m^1^A-generating methyltransferase complex TRMT6/61A and m^1^A modification of RNAs accumulate in SGs during heat shock stress (Alriquet et al., 2020).

Altogether, these novel studies point to RNA chemical modifications as a potential mechanism to finely tune the recruitment of RNAs and their associated proteins to SGs. This, together with the specific conformations of RNA molecules recruited into SGs, might control the assembly and disassembly of granules, and contribute to the organization of macromolecules inside SGs and the regulation of their functions.

## Stress Granules as Therapeutic Targets in RNA Virus Infection, Cancer and Neurodegeneration

Stress granule dysfunction has been linked to viral infections, cancer, and neurodegenerative diseases among others and, as such, have been proposed as targets for treatment of a broad-spectrum of diseases. In this section, we are going to briefly review how SGs are involved in different pathological states and how we could use SGs as targets for medical interventions. For further reading, extensive reviews on this subject are referenced at the beginning of each subsection.

### Viral Infection

Substantial evidence indicates that RNP granules and viruses are intimately connected (McCormick and Khaperskyy, 2017; Gaete-Argel et al., 2019; Zhang Q. et al., 2019; Eiermann et al., 2020). Viruses induce stress on the host cells and the formation of SGs, which are a crucial component of the cellular anti-viral response. Because viruses depend on the host translation machinery, RNA and DNA viruses need to counteract SG formation. A popular strategy among RNA viruses is interfering with SG assembly by using viral RBPs to block the activity of PKR, one of the cellular stress sensors. For example, the RBP accessory protein 4a of Middle East respiratory syndrome coronavirus (MERS-CoV) impedes dsRNA-mediated PKR activation, inhibiting eIF2α phosphorylation and SG formation (Rabouw et al., 2016). Another strategy of RNA viruses is interfering with SG assembly by sequestering, redistributing, or cleaving nucleating proteins of SGs (Zhang Q. et al., 2019). For example, the 3′ stem-loop of the West Nile virus (WNV) captures TIA-1 and TIAR SG proteins facilitating virus replication (Li et al., 2002), and severe acute respiratory syndrome coronavirus 2 (SARS-CoV-2) nucleocapsid (N) protein interacts with G3BP1/2 to disassemble SGs and facilitate viral production (Luo et al., 2021). Furthermore, cleavage of G3BP1 by poliovirus 3C proteinase is a strategy the poliovirus uses to inhibit SG formation (White et al., 2007). Interestingly, the picornavirus EV71 induces only atypical SGs (aSGs) through the cleavage of eIF4GI by 2A protease. These aSGs are beneficial to viral translation through sequestering only cellular mRNAs, but not viral mRNAs (Yang et al., 2018).

Recently, bioinformatic analyses proposed that the N protein of SARS-CoV-2, which contains low complexity domains (LCD) and a high tendency to phase separate, is capable of forming or regulating RNA granules through interactions with host SG-RNAs and proteins (Cascarina and Ross, 2020; Moosa and Banerjee, 2020). A series of different studies showed that the N protein does phase separate with RNA and other proteins such as hnRNPs, RNA polymerase, and membrane-associated M protein (Perdikari et al., 2020; Savastano et al., 2020; Lu et al., 2021). Importantly, phase separation is driven by genomic RNA elements and regulated by phosphorylation of N protein and ATP (Carlson et al., 2020; Iserman et al., 2020; Dang et al., 2021).

On the other side, many viruses induce the formation of intracellular compartments in the host cell called viral factories, structures that concentrate nucleic acids and viral proteins, as well as some specific cellular factors. Viral factories facilitate viral replication and assembly and protect the viral genome from the cell defense mechanisms. Interestingly, some viruses form nuclear or cytoplasmic membraneless viral factories that have liquid organelle properties (Nevers et al., 2020). An example of these structures are viroplasms of the Reoviridae family of viruses, which are nucleated by viral non-structural proteins NSP2 and NSP5 in rotaviruses (Eichwald et al., 2012) and inclusion bodies of measles virus, whose phase separation is triggered by nucleoprotein and phosphoprotein of the virus (Zhou et al., 2019). Ebola virus, which also induces the formation of inclusion bodies for its replication and transcription, needs host nuclear RNA export factor 1 (NXF1), an important component of the nuclear mRNA export pathway. NXF1 interacts with Ebola virus nucleoprotein and then with viral mRNAs in inclusion bodies and probably assists with the export of viral mRNAs to ribosomes for translation (Wendt et al., 2020).

Interestingly, it has been demonstrated that a synthetic double-stranded (ds)RNA that mimics viral infection, polyinosinic–polycytidylic acid [poly(I:C)], induces persistent formation of mutant FUS/TLS SGs. FUS/TLS is an RNA-/DNA-binding protein that forms pathological inclusions in ALS, and mutations in *FUS* gene are associated to ALS (Kwiatkowski et al., 2009; Vance et al., 2009; Mejzini et al., 2019). Poly(I:C) induces FUS-enriched cytoplasmic assemblies in a mechanism that is enhanced by type I interferon (IFN), the central component of antiviral signaling (Shelkovnikova et al., 2019).

**FIGURE 3:**
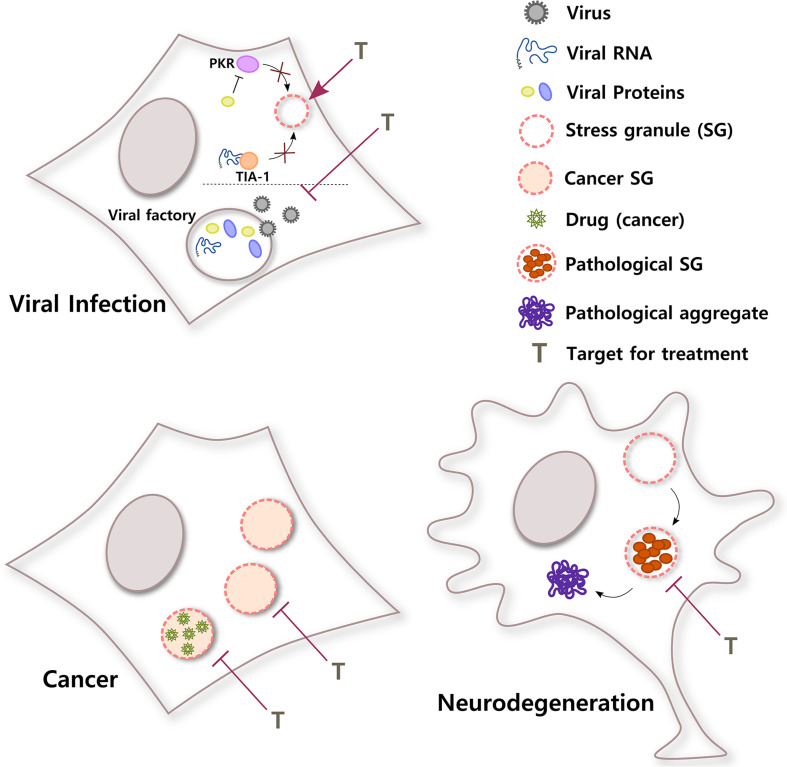
SGs in different diseases. SGs have important roles in a broad variety of pathological stages. In viral infections, viruses use different strategies to counteract the antiviral response of SGs in the cell. For example, viral RNA can sequester T-cell-restricted intracellular antigen-1 (TIA-1) to inhibit SG formation or a viral protein can block PKR, a kinase that senses environmental stress. In addition, some viral factories, sites of replication, and packaging inside the cell have liquid organelle properties. Both protection of host SGs and destabilization of viral factories could be potential targets for viral infection. Conversely, cancer cells can induce selective translation of stress-adaptive mRNAs and SG formation to promote survival, chemoresistance, and metastasis. Interestingly, SGs also concentrate antineoplastic drugs, altering drug pharmacodynamics. Inhibiting the formation of SGs in cancer cells or the partitioning of cancer drugs into SGs might improve their efficacy. In neurodegenerative diseases such as ALS, chronic stress and/or mutations in SG proteins could induce the formation of rigid complexes in SGs, turning them into pathological structures that could be precursors of pathological aggregates. Treatments that selectively target pathological SGs might help to reduce the formation of irreversible toxic aggregates and improve neuronal survival.

In summary, evidence indicates that RNA viruses not only need to inhibit SG assembly in order to successfully accomplish the different steps of the virus cycle and generate progeny, but many viruses also need to build membraneless replication compartments, which share many characteristics with SGs. The development of treatments that shield host SGs and guarantee their formation during viral infections or that specifically destabilize viral factories without affecting other RNA granules in the cell will provide so necessary broad-spectrum antivirals (Figure 3).

### Cancer

Extensive reviews have been published regarding the links that exist between SGs and cancer (El-Naggar and Sorensen, 2018; Gao et al., 2019; Spannl et al., 2019; Verdile et al., 2019; Aulas et al., 2020). In brief, first, SG components are involved in carcinogenesis and cancer metastasis. For example, all FET RBPs—fused in sarcoma/translocated in liposarcoma (FUS/TLS), Ewing sarcoma breakpoint region 1 (EWS), and TATA-box-binding protein-associated factor 15 (TAF15)—localize into SGs and participate in cancer. FUS/TLS has been identified forming fusion with different genes such as DNA damage-inducible transcript 3 protein (*DDIT3* or *CHOP*) and V-Ets avian erythroblastosis virus E26 oncogene homolog (*ETS2*), and several rearrangements between domains of different transcription factors and the RNA-binding domain of *EWSR1* or the N-terminal domain of *EWSR1* or *TAF15* have been reported in cancer (Campos-Melo et al., 2014). Moreover, several SG proteins that participate in the formation and regulation of SGs have been reported to be involved in cancer. For example, the reduction in TIA-1 and TIAR has been shown to trigger cell proliferation and tumor growth and accelerate mitotic entry, respectively (Sanchez-Jimenez et al., 2015; Lafarga et al., 2019). However, a short splicing variant of TIA-1 expressed in human colon cancer has been shown to exert the opposite effects by enhancing tumor growth, angiogenesis, and chemoresistance, adding complexity to the participation of SG proteins in cancer (Hamdollah Zadeh et al., 2015; Aulas et al., 2020). G3BP1, another RBP critical for SG assembly, has been shown to promote tumor progression, metastasis, cell proliferation, and chemoresistance (Dou et al., 2016; Wang Y. et al., 2018; Zhang Q. et al., 2019; Zhang C. H. et al., 2021; Zhao et al., 2021).

Second, SGs induce survival in certain tumors. Cancer cells initially depend on the local environment for growing. At a certain point, the tumor outgrows the local blood supply, generating regions of reduced nutrients and oxygen levels, and high concentration of reactive oxygen species (ROS). These and other stressors could be lethal for tumor cells unless they rapidly adapt through stress-resilient clones (Anderson et al., 2006; Quaranta et al., 2008; Ruggero, 2013). Emergent evidence suggests that one mechanism of clonal selection and acquisition of aggressive phenotypes (chemoresistance and metastatic capacity) is through selective translation of stress-adaptive mRNAs that encode for tumor cell survival proteins (Leprivier et al., 2013, 2015; El-Naggar et al., 2015). An efficient response of cancer cells to stress occurs through the formation of SGs, the correct exclusion of cancer essential mRNAs from SGs, and the reprogramming of translation (Somasekharan et al., 2015; Grabocka and Bar-Sagi, 2016; El-Naggar and Sorensen, 2018). In addition, several cancer drugs have been reported to induce the formation of SGs and, thus, resistance to chemotherapy-induced apoptosis (Anderson et al., 2015). Importantly, for a long time, it was assumed that cancer drugs get equally distributed in the cytoplasm of cells. However, we now know that specific membraneless organelles of tumor cells concentrate antineoplastic drugs and that this partitioning influences drug activity, suggesting that this phenomenon contributes to the cancer drug pharmacodynamics (Klein et al., 2020).

Third, there is evidence that interference with the dynamic of SGs negatively impacts on cancer. Knocking-down G3BP1 reduces SG assembly in cancer cells treated with bortezomib (BZM), a proteasome inhibitor used for a range of hematological tumors, potentiating chemotherapeutic-induced cancer cell death (Klein et al., 2020). Furthermore, a small molecule called C108 that binds G3BP2, increases CD8 T-cell proliferation and infiltration, as well as survival and long-term cures in breast tumor-bearing mice (Zhang Y. et al., 2021). Last, in glioblastoma cells, raloxifene, an estrogen receptor modulator, prevents SG dissolution, impairs protein synthesis control, and promotes cell death during hypoxia. Then, modulating SGs could be used to exploit the hypoxic niche of glioblastoma tumors (Attwood et al., 2020).

Altogether, these data suggest that inhibiting SG assembly and changing the stress conditions of the tumor can influence tumor progression and sensitize cancer cells to chemotherapeutic agents, representing a promising strategy for cancer treatment. It also suggests that because drugs selectively partition into membraneless organelles, cells can develop drug resistance through a mechanism that involves condensates (Figure 3). This should be taken into consideration for the design of efficient drugs to treat different diseases.

### Neurodegenerative Diseases

RNA-binding proteins that localize in SGs such as TIA-1, TDP-43, and G3BP1 have been observed in pathological aggregates of neurodegenerative diseases like Alzheimer’s, Huntington’s and ALS. These findings suggest that SG dysregulation might have a role in protein aggregate formation in neurodegeneration (Ash et al., 2014; Vanderweyde et al., 2016; McAleese et al., 2017; St-Amour et al., 2018; Coudert et al., 2019; Ryan and Fawzi, 2019; Wolozin and Ivanov, 2019; Dudman and Qi, 2020).

One of the strongest hypotheses in neurodegeneration is that SGs could be precursors of pathological aggregates in diseases such as ALS and FTD. It has been proposed that chronic stress and aging could alter the composition of SGs in a way that favors the formation of rigid complexes that grow up to insoluble aggregates, altering the physiology of the cells. Evidence from different avenues points to the connection between SGs and neurodegeneration in ALS/FTD. Besides the localization of SG RBPs in pathological aggregates, mutations in genes that encode for these proteins have been reported in ALS/FTD patients. For example, mutations in TIA-1, hnRNPA1, FUS/TLS, and TDP-43 have been described in ALS/FTD (Benajiba et al., 2009; Kwiatkowski et al., 2009; Pesiridis et al., 2009; Gendron et al., 2013; Kim et al., 2013; Mackenzie et al., 2017; Baradaran-Heravi et al., 2020). Furthermore, the expression of poly(GR) dipeptide repeat proteins in mouse brain, the product of G_4_C_2_ repeat expansions and the most common genetic cause of ALS and FTD, has been shown to induce the formation of SGs that colocalize with aggregated poly(GR) (Zhang et al., 2018).

*In vitro* experiments have shown that liquid-like droplets of SG proteins, such as FUS/TLS and TDP-43, convert into an aggregated state in time and that this conversion is accelerated by disease-associated mutations (Murakami et al., 2015; Patel et al., 2015; Gasset-Rosa et al., 2019). Importantly, *in vivo* studies using a light-inducible SG system based on optogenetic multimerization of G3BP1 allowed to demonstrate that persistent formation of SGs is cytotoxic. These SGs evolve in time to neuronal pathological inclusions characteristic of ALS/FTD (Zhang P. et al., 2019). The arrangement of LCDs of several RBPs into kinked β-sheet structures that interact weakly through polar atoms and aromatic side chains, and pair into protofilaments, could explain the accumulation of insoluble proteins in pathological tissue (Hughes et al., 2018). Similarly, two-photon imaging in a *FUS/TLS* knock-in ALS mice model transduced with TIA-1-EGFP have shown intense TIA-1-EGFP-positive granules formed in the cortex neurons in hours but cleared weeks after stress challenge. Neurons showing severe granule misprocessing die days after stress challenge, demonstrating that SG dysregulation is pathogenic in ALS (Zhang et al., 2020). Finally, studies in a *C9orf72* ALS mice model have shown that G_4_C_2_ repeats induce the formation of aberrant SGs and phosphoTDP-43 pathological inclusions (Todd et al., 2020).

A general consequence of the formation of pathological SGs is the alteration of the normal function of these granules and the sequestration of specific RBPs, compromising the function of these proteins and altering several cellular processes (Orru et al., 2016; Gordon et al., 2019). For example, proximity labeling proteomics approaches using ALS-associated C9orf72 dipeptides have shown alterations in disassembly engaged protein composition suggesting that they also could be relevant for ALS/FTD pathogenesis (Marmor-Kollet et al., 2020).

Motor neurons might be particularly vulnerable to SG dysregulation and pathological aggregate formation (Wolozin and Ivanov, 2019). The reason for this vulnerability is still unclear, but it could be related to differences in the composition of RNAs and/or proteins of SGs. RNA and protein species in SGs as well as chemical modifications, structures, and interactions of these molecules might be different in motor neurons under stress than in other neuronal cells and then make these SGs susceptible to pathological organization. Size and morphology of motor neurons could also contribute to the formation of pathological SGs. Motor neurons have large somas and long axons, which imposes extraordinarily high energetic requirements to maintain, for example, RNA and protein homeostasis and manage cellular stress. Moreover, poor solubility of proteins that are prone to aggregation in motor neurons might contribute to the formation of pathological aggregates (Ciryam et al., 2017; Yerbury et al., 2019).

Even though other lines of investigation suggest that there could be more than one mechanism for pathological aggregate formation in neurodegeneration (Ambadi Thody et al., 2018; Droppelmann et al., 2019; Almeida and Brito, 2020; Dominguez-Meijide et al., 2020; Mahul-Mellier et al., 2020), today, there is a considerable amount of evidence that supports the idea of pathological SGs as a nidus for protein aggregation in neurodegeneration (Figure 3). Both *in vitro* and *in vivo* studies are quickly generating extremely valuable knowledge that will be the foundation to develop therapeutic approaches to prevent the transition from dynamic SGs to irreversible inclusions in these devastating diseases.

## Discussion

The emergence of the role of the RNA in RNP granule architecture is creating a more complete picture of the functioning of these dynamic organelles. Now, we know that not only specific proteins are necessary for the assembly of SGs but also RNA molecules with special characteristics. RNA is able to phase separate in the absence of proteins and work as efficient scaffolds for protein complexes. Moreover, the RNA structure drives interaction with proteins. In this review, we propose that under stress conditions, RNA and/or enzymes that chemically modify RNA could bind metabolites and ions, triggering changes in the structure and local charges of RNA molecules that prompt RNA–RNA interactions, RNA phase separation, and SG nucleation, contributing to the composition and organization of these RNP assembles (Figure 2).

Our knowledge to this point is still limited, and there are still important questions to be answered. How are RNAs selected to phase separate and nucleate SGs if there is no apparent specificity in the process and any RNA could potentially partitionate into these organelles? If there are RNA destination signals to SGs, at which structural levels are they encoded, and do they depend on stress sensor properties of the RNA molecule? Are RNA molecules recruited into SGs in a stepwise process? Finally, are RNAs subcompartmentalized in different domains and contain specific functions inside SGs? The elucidation of these and other interesting questions will allow us to move several steps forward in the understanding of the function of RNA in these dynamic structures.

Even though *in vitro* reconstitution of condensates and cell culture lines have been extremely useful in understanding the dynamic of RNP granules, these systems also have well-recognized limitations (Roden and Gladfelter, 2020). *In vivo* experiments in yeast and mice are starting to provide invaluable information regarding the physiological relevance of *in vitro* findings and their connection with pathological stages (Zhang P. et al., 2019; Sathyanarayanan et al., 2020; Todd et al., 2020; Zhang et al., 2020). Recently, live-cell imaging in *S. cerevisiae* has determined that during glucose deprivation, proteins are sequestered into SG in a process triggered by ATP exhaustion. In this study, Hsp104 chaperone ATP hydrolysis activity was demonstrated to determine aggregate dissolution and protein aggregate steady-state size (Sathyanarayanan et al., 2020).

Together with *in vivo* studies of the dynamic of SG formation, we anticipate that advances in the RNA structural field are going to open enormous possibilities to understand different RNP granules and discover new roles for RNA molecules. Recently, two techniques have been developed to study RNA structure. Single-particle cryo-electron microscopy has been used together with M2-seq biochemical analysis and computer modeling to determine 3D models of 11 different RNA molecules (Kappel et al., 2020), and dimethyl sulfate mutational profiling with sequencing (DMS-MaPseq) has been used to reveal alternative conformations of the same RNA sequence (HIV-1 RNA) in cells (Tomezsko et al., 2020). Although resolution and RNA sequence length are still constraints of these techniques, they have made crucial progress in the field.

RNA molecules have been increasingly recognized as potential therapeutic targets for different diseases, not only because by targeting mRNAs, functions of proteins that are very difficult to drug or undruggable might be modulated, but also because the vast majority of the human genome encodes for non-coding RNAs (Warner et al., 2018; Shao and Zhang, 2020; Umuhire Juru and Hargrove, 2020). Investigating RNA species and their structures and functions in SGs, as well as developing new small molecules and oligonucleotides that target specific RNAs in these granules, will allow us to finely modulate SG assembly and disassembly. RNP granules are an efficient transient organization of the cells, adaptable for many different uses. That could be the reason why SGs, and probably other dynamic assembles, are involved in a diverse group of diseases. The similarity in the conformation of different RNA granules might be a large strength if we are willing to develop therapeutic approaches for different pathological states.

## Author Contributions

DC-M, ZH, and MS conceptualized the study. DC-M prepared the original draft. DC-M, ZH, CD, and MS wrote, reviewed, and edited the manuscript. All authors have read and agreed to the published version of the manuscript.

## Conflict of Interest

The authors declare that the research was conducted in the absence of any commercial or financial relationships that could be construed as a potential conflict of interest.
